# Antigen 5 Allergens of Hymenoptera Venoms and Their Role in Diagnosis and Therapy of Venom Allergy

**DOI:** 10.1007/s11882-020-00954-0

**Published:** 2020-07-09

**Authors:** Simon Blank, Murilo Luiz Bazon, Johannes Grosch, Carsten B. Schmidt-Weber, Márcia Regina Brochetto-Braga, Maria Beatrice Bilò, Thilo Jakob

**Affiliations:** 1 Center of Allergy and Environment (ZAUM), School of Medicine and Helmholtz Center Munich, German Research Center for Environmental Health, Member of the German Center of Lung Research (DZL), Technical University of Munich, Ingolstädter Landstraße 1, 85764 Munich, Germany; 2 Department of General and Applied Biology, Biosciences Institute, Sao Paulo State University, Rio Claro, São Paulo Brazil; 3 Department of Clinical and Molecular Sciences, Ancona and Allergy Unit, Department of Internal Medicine, University Hospital of Ancona, Polytechnic University of Marche, Ancona, Italy; 4 Experimental Dermatology and Allergy Research Group, Department of Dermatology and Allergology, Justus-Liebig-University Gießen, Giessen, Germany

**Keywords:** Antigen 5, Component-resolved diagnostics, Allergen cross-reactivity, Hymenoptera venom allergy, *Polistes dominula* venom, Yellow jacket venom

## Abstract

**Purpose of Review:**

Stings of Hymenoptera of the superfamily Vespoidea such as yellow jackets, paper wasps or stinging ants are common triggers for severe and even fatal allergic reactions. Antigen 5 allergens are potent allergens in the majority of these venoms with major importance for diagnosis and therapy. Reviewed here are the characteristics of antigen 5 allergens, their role in component-resolved diagnostics as well as current limitations of the available diagnostics for proper therapeutic decisions.

**Recent Findings:**

Antigens 5 are proteins of unknown function in Hymenoptera venoms with high allergenic potency. They represent key elements in component-resolved diagnosis to discriminate between honeybee and vespid venom allergy. However, due to their pronounced cross-reactivity, there are remaining diagnostic and therapeutic challenges that have to be addressed.

**Summary:**

Antigens 5 are highly relevant venom allergens of the Vespoidea superfamily. Although their use in component-resolved diagnosis facilitates dissection of cross-reactivity and primary allergy in double sensitization to honeybee and vespid venom, new diagnostic concepts are needed to discriminate between allergies to different vespid species.

## Introduction

Stings of Hymenoptera are one of the most frequent triggers for severe IgE-mediated anaphylaxis in adults [1]. Systemic reactions to the venoms of stinging Hymenoptera may be restricted to generalized symptoms of the skin, but can also affect the respiratory and vascular system and lead to multiorgan failure. Fatal anaphylaxis after Hymenoptera stings is a rare but well-recognized cause of sudden death [2] and accounts for approximately 20% of cases of anaphylaxis-related fatalities [3]. Hymenoptera venom allergy can be effectively treated by venom-specific allergen immunotherapy (VIT), which represents the only available curative treatment. Efficacy and safety of VIT highly depend on the unequivocal identification of the culprit insect causing clinical symptoms and, hence, the correct choice of venom for therapy. VIT was reported to be effective in preventing subsequent systemic sting reactions in 77–84%, 91–96% and 97–98% of patients allergic to honeybee venom (HBV), yellow jacket venom (YJV) and ant venom, respectively [4].

Allergy-relevant Hymenoptera belong to the superfamilies of Apoidea and Vespoidea. Honeybees (*Apis mellifera*) are elicitors of venom allergy in areas all over the world and also yellow jackets (*Vespula* spp.) are common allergy-relevant species, particularly in the Northern hemisphere, whereas paper wasps are of greater importance in the US (e.g. *P. annularis*, *P. exlamans*) and the Mediterranean region of Europe (*Polistes dominula*). In South America, other Polistinae such as *Polybia*, *Agelaia* and *Apoica* are of special importance. Moreover, venom allergy can be caused by stings of bumblebees (*Bombus* spp.) and hornets (*Vespa* spp., *Dolichovespula* spp.). Allergic reactions to the venoms of stinging ants are of major relevance in North and South America (fire ants; *Solenopsis* spp.), Australia (jumper ant; *Myrmecia pilosula*) and Asia (Asian needle ant; *Pachycondyla chinensis*).

In addition to Phospholipase A1 (PLA1) [5], antigen 5 (Ag5) represents one of the most important major venom allergens in almost all allergy-relevant Vespoidea species [6–12]. Only for *Myrmecia pilosula*, no Ag5 was annotated as allergen so far. Although Ag5 allergens are the most abundant proteins in most Vespoidea venoms, their function within the venoms remains largely unclear [13].

In recent years, the focus in venom allergy research has increasingly shifted from whole venoms to individual allergenic molecules. This had led to the development of component-resolved diagnostics (CRD) [14•, 15•, 16•, 17–19], which uses single allergens of the venoms instead of whole venom extracts to measure specific IgE (sIgE) antibodies in patients’ sera. Particularly, for the differentiation between cross-reactivity and primary sensitization to HBV and YJV, CRD with species-specific marker allergens such as Ag5 (Ves v 5) and phospholipase A2 (Api m 1) added value. However, due to the high degree of cross-reactivity between the major allergens of vespid venoms, discrimination of allergies to different vespid species such as yellow jackets (*Vespula* spp.) and paper wasps (*Polistes* spp.) remains challenging.

Due to their outstanding role as major allergens, Ag5 proteins build a key element for diagnosis of Vespoidea venom allergy. For instance, molecular diagnosis applying Ag5 (Ves v 5) of yellow jackets has already proven to be able to increase diagnostic sensitivity and has led to the development of advanced diagnostic tests [20••]. Nevertheless, there is an urgent need for new diagnostic concepts, which due to their relevance as allergens surely have to include Ag5 proteins, to dissect primary allergy and cross-reactivity in vespid venom allergy. Moreover, Ag5 proteins, as major sensitizing allergens of Vespoidea venoms, may represent a reliable basis for the design of new therapeutic strategies in Hymenoptera venom allergy.

## Antigen 5 Homologs in Different Species and Their Antigenic Cross-reactivity

**Fig. 1:**
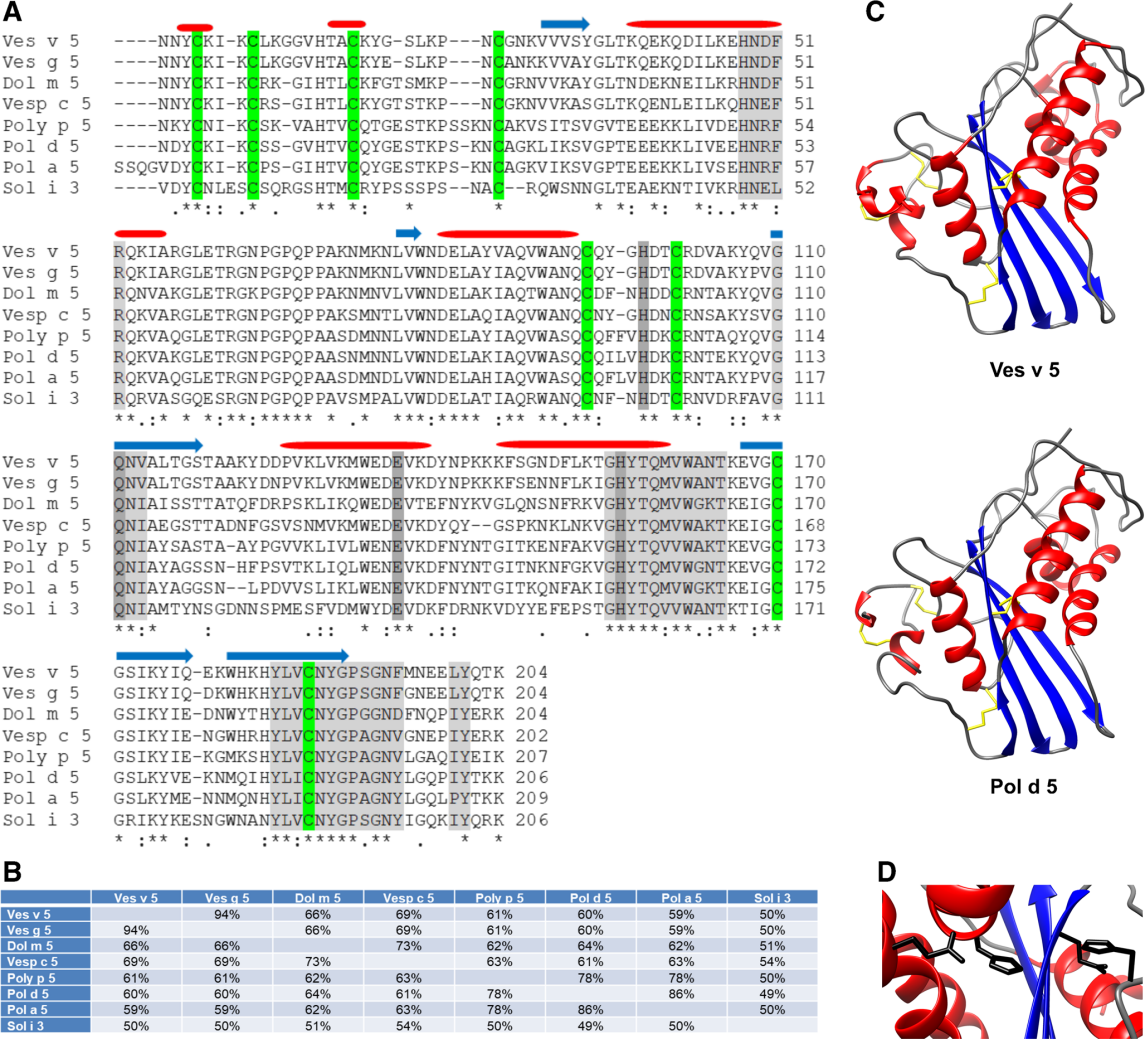
Antigen 5 homologues and their structure. **a** Alignment of the mature sequences of selected Hymenoptera antigen 5 allergens. The secondary structure elements identified in Ves v 5 are indicated above the relevant amino acid sequences (red, α-helix; blue, β-strand). These elements are conserved between the different Ag5 proteins. The CAP signature motifs (CAP3, CAP 4, CAP1, CAP2) and the typical motif [ILVP]Y, which is found near the terminus of Ag5 proteins, are marked in light grey. Conserved residues that form the putative active site are marked in dark grey. Cysteine residues that form disulphide bridges are marked in green. Asterisks, colons and periods indicate identical, conserved and semi-conserved residues, respectively. **b** Percent identity between the different antigen 5 allergens. Sequence identifiers: Ves v 5 (Q05110.1), Ves g 5 (CAJ28930.1), Dol m 5 (P10736.1), Vesp c 5 (P35781.1), Poly p 5 (P86686.1), Pol d 5 (NP_001310265.1), Pol a 5 (Q05109.1), Sol i 3 (XP_011165202.1). **c** Crystal structure of Ves v 5 (1QNX) [37] and structural model of Pol d 5 [32••]. α-helices, β-strands and coiled regions are shown in red, blue and grey, respectively. Disulphide bridge-forming cysteines are indicated in yellow. **d** The solvent-exposed cleft (Ves v 5), which contains the putative active site, formed by a conserved dihistidine motif and conserved residues (Glu, Gln) providing a supporting hydrogen bond network

Ag5 proteins of Hymenoptera belong to the CAP (cysteine-rich secretory proteins, antigen 5 and pathogenesis-related 1 proteins) superfamily, whose members are found in a wide range of organisms including plants as well as members of each of the animal kingdoms and are involved in diverse biological processes such as reproduction, cancer, immune regulation and host defense [13]. The Ag5 proteins form a major and distinct clade of the CAP superfamily and are mainly found in stinging and blood-feeding insects [13]. While most representatives of the CAP superfamily are secreted and function as endocrine or paracrine modulators, the role of Hymenoptera Ag5 proteins remains elusive [13]. In blood-feeding ticks, flies and mosquitoes, Ag5 proteins are part of a mixture of salivary proteins that are thought to function either in suppression of the host immune system or in preventing platelet aggregation [21]. This biological function is similar to that found for other CAP proteins, e.g. of parasitic nematodes or lampreys, and, therefore, most likely encoded within the CAP domain (Fig. 1a). The presence of Ag5 allergens (as well as hyaluronidases), which exhibit cross-reactivity with their homologues of wasp venom, in the salivary of horseflies and mosquitoes [22], may explain the postulated “wasp-mosquito-horsefly-syndrome”, in which wasp venom-allergic patients also experience systemic reactions after bites of mosquitoes or horseflies [23, 24].

Recently, an Ag5-like protein was also identified at transcriptomic level in the venom glands of winter but not of summer bees. However, the recombinantly produced protein showed neither IgG4 reactivity with sera of beekeepers nor cross-reactivity with YJV Ag5 (Ves v 5). This might be explained by a lack of sting exposure in the winter, low abundance in the venom and/or low sequence identity (approx. 25%) [25].

**Fig. 2:**
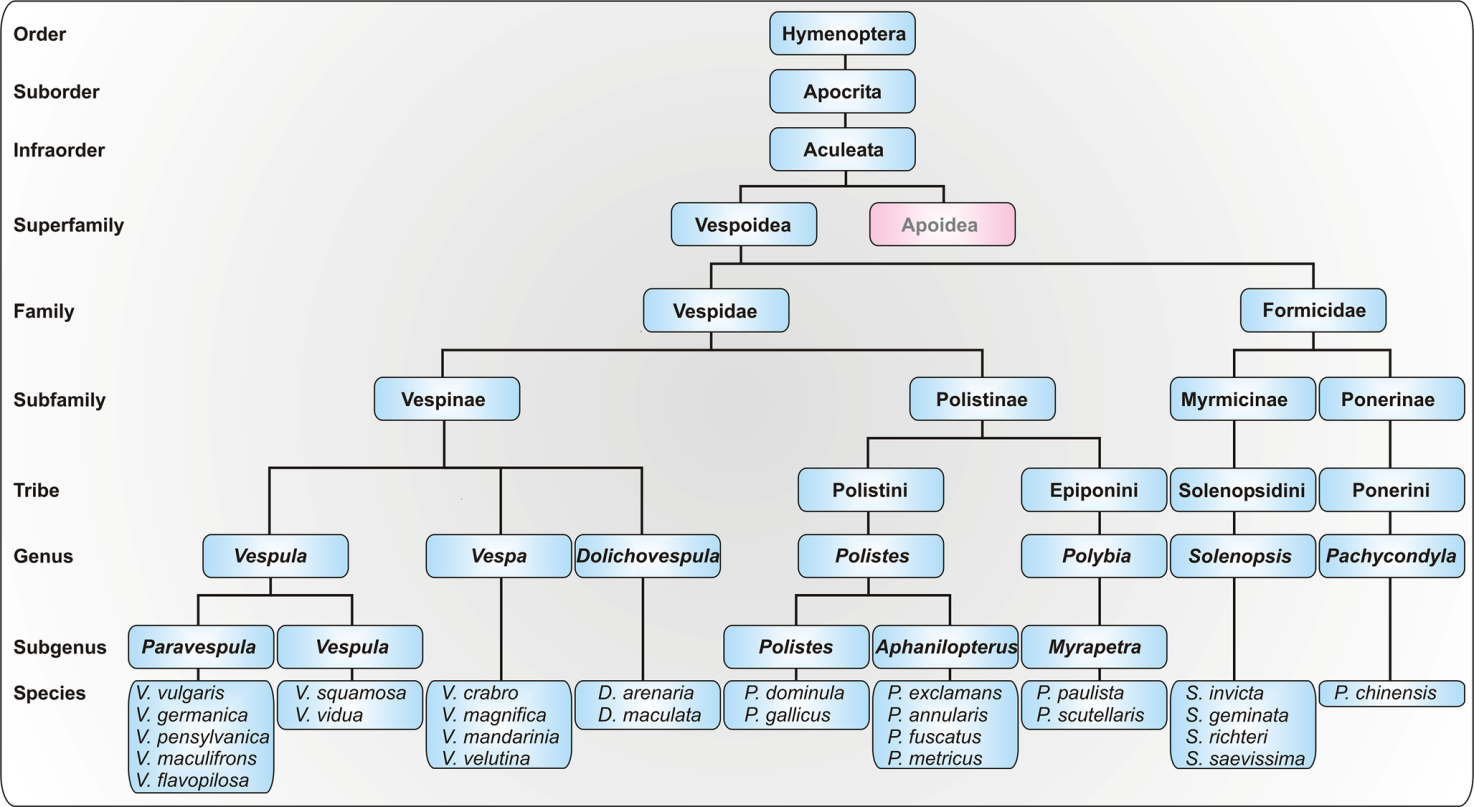
Taxonomy of Hymenoptera species for which an antigen 5 is annotated as allergen. Other allergy-relevant Hymenoptera species such as honeybees and bumblebees belong to the superfamily Apoidea

To date, 26 Vespoidea Ag5 proteins are listed as allergens (Table 1) in the official allergen nomenclature database of the World Health Organization and International Union of Immunological Societies (WHO/IUIS) [26]. An alignment of selected Hymenoptera Ag5 allergens is shown in Fig. 1a. According to the phylogenetic distance between these species (Fig. 2), the Ag5 allergens exhibit a varying degree of sequence identity (Fig. 1b) and, therefore, most likely of cross-reactivity.

**Table 1:** Antigen 5 allergens currently listed in the official WHO/IUIS allergen nomenclature database [26]

Species	Common name	Allergen	Sensitization rate^a^
*Dolichovespula arenaria*	Yellow hornet	Dol a 5	81%^b^
*Dolichovespula maculata*	White-faced hornet	Dol m 5	65% [7]
*Pachycondyla chinensis*	Asian needle ant	Pac c 3	83% [36]
*Polistes annularis*	American paper wasp	Pol a 5	44–65% [30•, 32••]^2^
*Polistes dominula*	European paper wasp	Pol d 5	72% [30•]
*Polistes exclamans*	American paper wasp	Pol e 5	90% [7]
*Polistes fuscatus*	Golden/Northern paper wasp	Pol f 5	69% [83]
*Polistes gallicus*	European paper wasp	Pol g 5	80–100% [84]
*Polistes metricus*	Metricus paper wasp	Pol m 5	Yes [83]^c^
*Polybia paulista*	Polybia wasp	Poly p 5	100% [57•]
*Polybia scutellaris*	Polybia wasp	Poly s 5	58–70% [32••]^c^
*Solenopsis geminata*	Tropical fire ant	Sol g 3	100% [85]
*Solenopsis invicta*	Red imported fire ant	Sol i 3	67% [9]
*Solenopsis richteri*	Black fire ant	Sol r 3	Yes [86]^c^
*Solenopsis saevissima*	Brazilian fire ant	Sol s 3	?
*Vespa crabro*	European hornet	Vesp c 5	67% [32••]^c^ Yes [87]
*Vespa magnifica*	Hornet	Vesp ma 5	73–91%^b^
*Vespa mandarinia*	Giant Asian hornet	Vesp m 5	?
*Vespa velutina*	Asian hornet	Vesp v 5	86%^b^
*Vespula flavopilosa*	Downy yellow jacket	Ves f 5	?
*Vespula germanica*	German yellow jacket	Ves g 5	Yes [88]
*Vespula maculifrons*	Eastern yellow jacket	Ves m 5	Yes [10]
*Vespula pensylvanica*	Western yellow jacket	Ves p 5	Yes [89]
*Vespula squamosa*	Southern yellow jacket	Ves s 5	79% [7]
*Vespula vidua*	Long/Widow yellow jacket	Ves vi 5	?
*Vespula vulgaris*	Common yellow jacket	Ves v 5	85–100% (Table 2)

A question mark indicates that no data was found. Yes means that sensitization was shown but data about sensitization rates is not given.

^a^Determined by different methods and with highly variable patient numbers

^b^Data obtained from the WHO/IUIS allergen nomenclature database

^c^Analyzed patient cohort is not entirely suitable to assess sensitization to the given allergen

Ag5 allergens (as well as other allergens) of different *Vespula* species display a very high degree of sequence homology and are thought to be nearly completely cross-reactive [27]. Although sequence identity between Ag5 allergens of members of the Vespinae (*Vespula* spp., *Vespa* spp., *Dolichovespula* spp.) is less, they still exhibit pronounced cross-reactivity. The clinical relevance of this cross-reactivity is reflected by the fact that patients primary sensitized to YJV can develop severe, and even fatal, anaphylaxis after a hornet sting and vice versa and, moreover, that hornet-allergic patients can be adequately treated with yellow jacket VIT [2, 28]. However, one study from Italy (where *Vespa crabro* extract is available for VIT) suggests that in patients with ascertained primary *Vespa* allergy, VIT with *V. crabro* venom would be more adequate, at least concerning the safety profile [29].

Cross-reactivity between Ag5 allergens of the Vespinae and Polistinae subfamilies was described to be less pronounced as within the same subfamily [8]. Nevertheless, double-positive sIgE test results to YJV and *Polistes dominula* venom (PDV) are frequently observed and represent a diagnostic challenge in areas where both species are endemic [30•, 31••, 32••]. Due to a partial cross-reactivity between the Ag5 allergens and other venom allergens of European (*P. dominula*, *P. gallicus*) and American (e.g. *P. annularis*, *P. exclamans*, *P. fuscatus*) *Polistes* species, which belong to different subgenera (Fig. 2), diagnosis and therapy should be performed with the venoms of endemic species [30•, 32••, 33, 34]. While in first studies cross-reactivity between red imported fire ant Ag5 (Sol i3) and vespid Ag5 allergens was described to be absent [8, 35], a later study demonstrated pronounced cross-reactivity [32••]. Accordingly, cross-reactivity between Ag5 of the Asian needle ant (Pac c 3) and YJV Ag5 (Ves v 5) was demonstrated [36]. The clinical relevance of this cross-reactivity is so far unclear.

In recent study, which applied recombinantly produced Ag5 allergens from seven allergy-relevant species (*Vespula vulgaris*, *Vespa crabro*, *Dolichovespula maculata*, *Polistes dominula*, *Polistes annularis*, *Polybia scutellaris* and *Solenopsis invicta*), pronounced sIgE cross-reactivity between all Ag5 allergens was demonstrated in cohorts of primary YJV- and PDV-sensitized patients [32••]. Moreover, robust effector cell activation of YJV-allergic patients by all Ag5 allergens was demonstrated in basophil activation test (BAT). The clinical relevance of this observation at a symptom-based level remains elusive, as most of the patients were only stung by one of the species.

## Structural Aspects of Hymenoptera Antigens 5

So far, the crystal structures of YJV Ag5 Ves v 5 and fire ant Ag5 Sol i 3 were solved [37, 38]. The secondary structure elements (Fig. 1a) are arranged in an α-β-α-sandwich fold consisting of a central antiparallel β-sheet surrounded on both sides by α-helices (Fig. 1c). The alignment in Fig. 1a shows that most of the structural elements in Ves v 5 can be expected in all Hymenoptera Ag5 proteins, resulting in an identical fold that is characteristic for all members of the CAP superfamily [13]. Hence, structural modeling of Hymenoptera Ag5 proteins results in very similar three-dimensional structures [32••], as depicted exemplarily for PDV Ag5 Pol d 5 in Fig. 1c. These similar structures with conserved surface areas but also differences in side-chain properties of exposed amino acids suggest the presence of conserved but also unique B cell epitopes [32••, 37, 38], explaining the different pattern of cross-reactivity between closely related and phylogenetically more distant members of the Ag5 family. The Ag5 structures are stabilized by a number of disulphide bridges (Fig. 1 a and c), which provide the thermal, pH and proteolytic stability of CAP proteins [13].

The fact that the Ag5 proteins, in contrast to other members of the CAP superfamily, consist only of the CAP domain, which is characterized by 4 consensus sequences (CAP1–4) (Fig. 1a), implies that their function must be encoded within this domain [13]. Of note, 4 conserved amino acids, including 2 histidines (Fig. 1a), are solvent exposed and located in an elongated cleft (Fig. 1d). These residues are able to provide a hydrogen bond network and are believed to form a putative active site, perhaps with a bound divalent cation [13, 37].

## IgE Sensitization to Antigens 5

The rates of sIgE sensitization to Ag5 proteins that are annotated as allergens are depicted in Table 1. Of note, the sensitization rates to the different Ag5 allergens have been analyzed using a variety of methods, including immunoblotting, ELISA, effector cell activation tests and commercially available sIgE assay platforms. Moreover, inclusion criteria of patients as well as analyzed patient numbers, ranging from a few to hundreds, highly differ in the studies. Therefore, the obtained sIgE sensitization rates to the different Ag5 allergens are difficult to compare and a comprehensive picture of Ag5 sensitization in patients with primary allergy to the respective species cannot be drawn with absolute certainty. Nevertheless, the available studies suggest that Ag5 proteins most likely represent the most potent major allergens in all allergy-eliciting Vespoidea species, for which an allergenic Ag5 was identified.

Studies, addressing sensitization rates in large, well-defined patient populations on sIgE assay platforms, which are more standardized and more applicable for measurements in larger study cohorts, are currently only available for Ves v 5 from YJV. In a first study that used a no longer available liquid-phase detection system (ADVIA Centaur), sIgE to Ves v 5 was detected in 89/100 (89%) of patients with a history of YJV allergy and positive skin test to YJV. When the inclusion criteria were extended to patients with sIgE to YJV, 87/91 (96%) were positive to Ves v 5 [39]. In a following study using the ImmunoCAP™ system (Thermo Fisher Scientific, Uppsala, Sweden), Ves v 5-sIgE was found in 53/59 (90%) of patients with history of YJV allergy. Including only patients with detectable sIgE to YJV raised the sensitization rate to 94% (48/51), whereby no differences between YJV-monosensitized (ms) (94%) and YJV/HBV-double-sensitized (ds) (95%) patients were observed [40]. A follow-up study by Köhler et al. found sIgE to Ves v 5 in 158/170 (93%) of YJV-allergic patients with sIgE to YJV (92% and 94% in ms and ds patients, respectively) [41]. Using the same assay platform, Schiener et al. detected sIgE to Ves v 5 in 42/43 (98%) [32••] and Ebo et al. in 131/148 (89%) of patients with a history of YJV allergy; 90% (82/91) in YJV-ms patients and 92% (43/47) in patients ds to YJV and HBV or with discrepant YJV-sIgE and skin test results [42]. Comparably, in a large cohort of patients with YJV-allergy (including ms and ds patients), 277/308 (90%) were reactive to Ves v 5 [20••], whereas in another cohort of YJV-ms patients, the value was slightly lower with 85% (169/200) [43].

Interestingly, Selb et al. measured sensitization to Ves v 5 in the same patient population of YJV-ms patients using the ImmunoCAP™ and the Immulite™ (Siemens Healthcare Diagnostics, Eschborn, Germany) system and found sensitization rates of 82% (90/110) and 93% (102/110), respectively [44]. In another study, investigating 111 patients with YJV allergy, these results were confirmed. Here, the sensitization rates to Ves v 5 were 87% (96/111) and 92% (102/111) using the ImmunoCAP™ and the Immulite™ system, respectively [45]. However, the same study demonstrated a lower specificity for sIgE detection to Ves v 5 on the Immulite™ system (92% compared to 100% for the ImmunoCAP™ system). These differences in obtained sensitization rates, using the two sIgE assay systems mentioned above, are most likely not due to the quality of allergens used but rather to the difference in calibration approaches, resulting in an overestimation of sIgE levels in one system [46••, 47, 48]. An overview of sIgE sensitization rates to Ves v 5 in different study populations is given in Table 2 in the column “Sensitivity Ves v 5”.

**Table 2:** Diagnostic sensitivity of Ves v 5 and Ves v 1 in diagnosis of patients with a history of yellow jacket venom allergy

Patients	Sensitivity YJV	Sensitivity Ves v 5	Sensitivity Ves v 5/Ves v 1	Method^a^	Remarks	Reference
100	91% (91)^b^	89% (89)	n.d.	ADVIA Centaur^d^		[39]
91	IC sIgE	96% (87)	n.d.			
59	86% (51)^b^	90% (53)	n.d.	ImmunoCAP		[40]
32	IC sIgE	94% (30)	n.d.		ms	
19	IC sIgE	95% (18)	n.d.		ds	
8	IC neg.^b^	63% (5)	n.d.			
22	IC sIgE	n.s.	100% (22)	ImmunoCAP	ms	[51]
200	IC sIgE	85% (169)	92% (184)	ImmunoCAP	ms	[43]
163	n.s.	92% (150)	96% (156)	ImmunoCAP		[50]
26	IC neg.^b^	65% (17)	65% (17)			
308	83% (257)^b^	90% (277)	96% (296)	ImmunoCAP		[20••]
308	97% (298)^c^	90% (277)	96% (296)			
148	89% (131)^b^	89% (131)	94% (139)	ImmunoCAP		[42]
91	IC sIgE	90% (82)	98% (89)		ms	
17	IC neg.^b^	71% (12)	71% (12)			
170	IC sIgE	93% (158)	n.d.	ImmunoCAP		[41]
103	IC sIgE	92% (95)	n.d.		ms	
67	IC sIgE	94% (63)	n.d.		ds	
43	IC sIgE	98% (42)	n.d.	ImmunoCAP		[32••]
111	100% (111)	87% (96)	98% (109)	ImmunoCAP		[45]
111	98% (109)	92% (102)	n.d.	Immulite		
110	IC sIgE	82% (90)	90% (99)	ImmunoCAP	ms	[44]
110	IC sIgE	93% (102)	97% (107)	Immulite/ImmunoCAP	ms	
25	92% (23)^b^	92% (23)	100% (25)	Immulite		[49]
49^e^	88% (43)^b^	86% (42)	92% (45)			

*n.d.* not determined, *n.s*. not shown, *IC sIgE* sIgE to YJV ≥ 0.35 kU_A_/L was inclusion criterion, *IC neg.* sIgE to YJV < 0.35 kU_A_/L was inclusion criterion, *ms* yellow jacket venom-monosensitized, *ds* yellow jacket venom and honeybee venom-double-sensitized

^a^Values ≥ 0.35 kU_A_/L were considered positive

^b^YJV extract not spiked with Ves v 5 was used for measurement

^c^YJV extract spiked with Ves v 5 was used for measurement

^d^No longer available

^e^Patients with mastocytosis and/or elevated baseline serum tryptase

## Antigens 5 in Routine Allergy Diagnosis and Their Diagnostic Sensitivity

Routine diagnosis of Hymenoptera venom allergy is based on a combination of clinical history of a systemic sting reaction and the proof of sensitization by skin testing and/or in vitro measurement of venom-specific IgE antibodies. In recent years, CRD of Hymenoptera venom allergy rapidly evolved [14•, 15•, 16•, 17–19]. In contrast to extract-based sIgE diagnosis that measures sIgE levels to native whole venom extracts, levels of sIgE to single allergens of the venoms are determined in CRD. Thus, CRD not only provides information on whether a patient has sIgE to the whole venom, but also which allergens of the venoms are relevant for sensitization. Sensitization profiles obtained in this way can help to discriminate between cross-reactivity and primary sensitization to different venoms. This particularly holds true for vespid venom and HBV allergy since marker allergens, specific for the respective venoms, exist. Additionally, allergens for CRD can be recombinantly produced without cross-reactive carbohydrate determinants (CCDs). Hence, in contrast to venom extract-based diagnosis, clinically irrelevant sensitization to CCDs is excluded in CRD with recombinant CCD-free allergens [15•, 16•]. So far, only the Ag5 allergens of YJV (Ves v 5) and PDV (Pol d 5) are available for routine CRD on various diagnostic platforms for either singleplex (Ves v 5: Thermo Fisher Scientific, Siemens Healthcare Diagnostics and Dr. Fooke Laboratories; Pol d 5: Thermo Fisher Scientific) or multiplex testing (Ves v 5 and Pol d 5: Euroimmun and Macro Array Diagnostics).

Conclusive data about the diagnostic sensitivity is currently available for Ves v 5 only. Using Ves v 5 alone for the diagnosis of patients with a history of YJV allergy, the diagnostic sensitivity ranges between 89% and 92% using the currently available diagnostic assay systems [20••, 40, 42, 45, 49, 50]. In patient cohorts, for which detectable sIgE to YJV was inclusion criterion, diagnostic sensitivity ranged between 82 and 98% [32••, 40–44]. Interestingly, the lowest values were found in patient cohorts monosensitized to YJV. This phenomenon might be explained by lower levels of sIgE to individual allergens in monosensitized patients, an effect that was also found in HBV-allergic patients and that might be explained by a more advanced state of allergic immune deviation in double-sensitized subjects [41]. As outlined before, for patient cohorts that were analyzed using the ImmunoCAP™ and the Immulite™ system, higher diagnostic sensitivity (but lower specificity) was obtained with the Immulite™ system. An overview about diagnostic sensitivity using Ves v 5 for diagnostic work-up in YJV-allergic patients is given in Table 2. The sensitivity of Pol d 5 for the diagnosis of PDV allergy is hard to assess, as a high percentage of the respective patient populations is double-sensitized to PDV and YJV with unknown primary sensitizer.

PLA1 (Ves v 1) was the second YJV major allergen that was introduced for routine diagnosis of YJV allergy (ImmunoCAP™ system). The use of the combination of both major allergens resulted in a sensitivity of 92%–100% for the diagnosis of YJV allergy [20••, 42–45, 49–51]. Hence, the addition of Ves v 1 to Ves v 5 increased diagnostic sensitivity by an additional 4% to 11% in the different study populations (Table 2). However, a small percentage of YJV-allergic patients cannot be diagnosed using the commercially available YJV allergens. So far, it remains elusive if this diagnostic gap can be filled by other YJV allergens, such as Ves v 2 or Ves v 3.

It is important to note that the levels of sIgE to whole venom extracts or to individual venom allergens do not correlate with the severity of the sting reaction [43, 44, 52•]. Although no correlation between the number of recognized allergens of a venom and the severity of the sting reaction can be observed in clinical routine, detailed studies addressing this are still missing.

Interestingly, a study that addressed sensitization profiles of YJV-allergic patients with mastocytosis and/or elevated basal serum tryptase found sensitization to Ves v 5 in 42/49 (86%) of patients and the addition of Ves v 1 increased diagnostic sensitivity to 92% (45/49) using the Immulite™ system (research prototype allergen assays) [49]. In this high-risk patient group, diagnostic sensitivity could be increased to 100% only by Ves v 1- and Ves v 5-based CRD and lowering the threshold to 0.1 kU_A_/L. For two of the patients with severe anaphylaxis, who exhibited negative intracutaneous skin tests and YJV-sIgE < 0.1 kU/L, this was the only way to verify sensitization [49]. Other authors confirmed an improved diagnostic sensitivity in YJV-allergic patients with mastocytosis by lowering the threshold to 0.17 kU_A_/L, while good specificity was retained [53]. It was demonstrated before that sIgE levels between 0.1 and 0.35 kU_A_/L can be measured with high accuracy on the major singleplex sIgE immunoassay platforms and should be considered in the context of a clear clinical history of venom allergy, irrespective of the presence of mast cell disorders [54, 55].

Clear gaps exist for accurate diagnosis of allergy to neotropical wasps in South America. For instance, *Polybia paulista*, a species that is common in the southeast of Brazil, represents a neglected health problem and causes a large number of severe systemic and even fatal allergic reactions [56]. Here, no routine diagnostics is available so far, a fact, leading to challenges in identification of primary sensitization. Recently, also Ag5 (Poly p 5) and PLA1 (Poly p 1) were identified as targets of interest for diagnosis of *Polybia* venom allergy [57•, 58].

## Antigen 5-Spiked Venom Extracts for Diagnosis

When Ves v 5 became available for routine diagnosis, different studies demonstrated that sIgE to this allergen could be detected in 63% to 71% of patients with a history of YJV allergy but negative sIgE test results with YJV (Table 2) [40, 42, 50]. Following these observations, Vos et al. demonstrated in a population of 308 patients with confirmed YJV allergy that only 83% could be diagnosed with the conventional YJV ImmunoCAP™, while sensitization could be verified in 96% using the individual allergens Ves v 1 and Ves v 5 [20••]. Of the extract-negative patients, only one was tested positive for Ves v 1, whereas 84% (42/51) were positive for Ves v 5. Moreover, and in contrast to Ves v 1, in the extract-positive patients, the levels of sIgE to Ves v 5 were substantially higher than to YJV extract. These results suggested a shortage of reactive Ves v 5 IgE epitopes in the diagnostic extract. As Ves v 5 is the most abundant protein in YJV, it can only be speculated about the reasons for this reduced immunoreactivity. Presuming that an underrepresentation in the venom extract is unlikely, possible explanations could be an inefficient coupling to the solid phase of the assay or a masking of IgE epitopes by natural ligands in the venom extract.

In the same study, sIgE reactivity of the patient cohort with a Ves v 5-spiked YJV ImmunoCAP™ was analyzed. Compared with the conventional, the Ves v 5-spiked ImmunoCAP™ yielded substantially higher sIgE values in Ves v 5-positive sera and diagnostic sensitivity increased from 83 to 97% (Table 2). No relevant differences in reactivity were observed in Ves v 5-negative sera. The increase in sensitivity was not accompanied by a change in specificity as demonstrated using sera of 51 HBV-allergic patients. In 18/19 skin test-negative YJV-allergic patients, sensitization could be verified using the Ves v 5-spiked YJV ImmunoCAP™. Together, the combination of skin tests and sIgE detection to Ves v 5-spiked YJV confirmed sensitization in 300/301 patients [20••]. Consequently, the new Ves v 5-spiked YJV ImmunoCAP™ was introduced in summer 2012.

The usefulness of the new test was then also demonstrated in a small study, in which 11 Ves v 5-reactive patients who were negative to the conventional test could be diagnosed using the new Ves v 5-spiked YJV immunoassay [42]. Moreover, a French multicenter study was performed on Ves v 5- and Pol d 5-spiked YJV and PDV ImmunoCAPs™, respectively [59]. Here, it was also demonstrated that the use of the Ag5-spiked venom extracts results in an improved diagnostic sensitivity, but also in higher numbers of double-positive test results. Moreover, it was shown that the measurement of sIgE to Ves v 5, Pol d 5 and Ves v 1 in sera without detectable sIgE to the spiked extracts results only in minimal diagnostic sensitivity improvements. The improved sensitivity of both, Ag5-spiked YJV and PDV ImmunoCAPs™, was also confirmed for a population of Japanese venom-allergic patients [60]. In contrast to the previous reports, one study found only a slight, not significant increase of sensitivity from 94% (106/113) to 96% (87/91) using the conventional and Ves v 5-spiked extract, respectively [61]. Moreover, a comparable decrease of specificity of both tests was observed with increasing levels of total IgE.

## Antigen 5 for the Discrimination Between YJV and HBV Allergy

Double-positive sIgE test results to YJV and HBV are frequently observed [39, 40, 51, 62]. These double-positive results may either reflect true primary sensitization to both venoms or may be caused by IgE directed against CCDs, which are present on most natural Hymenoptera venom allergens [63, 64] or to homologous allergens present in both venoms. In the first case, VIT with both venoms is recommended, while in the second scenario, VIT with the primary sensitizing venom is sufficient. As venom extract-based sIgE testing does not allow discrimination between cross-reactivity and primary sensitization to both venoms, double-positive results strongly hamper the choice of the correct venom for VIT or might even lead to unnecessary treatment with both venoms, particularly in patients who were not able to correctly identify the culprit insect.

Fortunately, molecular or component-resolved diagnostics with recombinantly produced, CCD-free species-specific marker allergens, which are present in either YJV (Ves v 1, Ves v 5) or HBV (Api m 1, Api m 3, Api m 4, Api m 10), has proven to be able to unequivocally identify primary sensitization to a given venom in many cases [14•, 15•, 16•, 17, 18]. Other allergens such as the hyaluronidases (Ves v 2 and Api m 2) or dipeptidyl peptidases (Ves v 3 and Api m 5) share similarities and, thus, exhibit a varying degree of cross-reactivity.

In a first study using Ves v 5 and Api m 1 on the no longer available ADVIA Centaur platform, reactivity to both allergens and, hence, primary allergy to both venoms, was confirmed in 34/63 (54%) of venom extract-double-positive patients, while in the others, reactivity to only one of the marker allergens was detected [39]. A following study measured sIgE to Ves v 5 and Api m 1 on the ImmunoCAP™ platform and confirmed that sIgE detection to both marker allergens allows reliable discrimination between primary allergy and cross-reactivity in patients double-sensitized to venom extracts [40]. For instance, primary sensitization to both allergens was found in only 24% (8/33) of CCD-negative patients with double-positive tests to venom extracts. Moreover, enhanced diagnostic utility of both marker allergens was also demonstrated for the Immulite™ platform [44]. Ves v 1 was the second YJV allergen that was introduced for CRD. It was found that sensitivity of the two YJV allergens is sufficient and that a positive sIgE test result with one of them is indicative for YJV VIT in patients who are double-positive to both venoms and for whom the culprit insect could not be identified [42, 51]. However, missing reactivity to Api m 1 does not necessarily exclude primary HBV allergy. As the detection of sIgE to this HBV major allergen is not always sufficient to distinguish YJV and HBV allergy [65], additional allergens of HBV (Api m 2, Api m 3, Api m 5, Api m 10) were introduced (ImmunoCAP™) that further increased diagnostic sensitivity of CRD [41]. Interestingly, Gattinger et al. demonstrated that the panel of Ves v 1, Ves v 5, Api m 1 and Api m 10 allowed the identification of the culprit venom in 98% (85/87) of patients sensitized to YJV and/or HBV with good agreement to skin testing [52•]. Contrary, another study questioned the ability of the available allergen panel to resolve double-sensitization, as 70% (69/98) of the patients double-sensitized to venom extracts were also double-sensitized with at least one allergen of YJV and HBV. A possible explanation was found in the unavailability of potentially cross-reactive allergens from both venoms for CRD [66••]. However, it is not clear to which extend this phenomenon might be caused by true primary sensitization to both venoms (47% and 13% of patients showed also double-positive and double-negative skin tests, respectively).

Although diagnostic sensitivity of the currently available allergen panel, particularly of HBV, is not 100%, CRD has clearly improved discrimination of primary allergy and cross-reactivity in YJV and HBV allergy and, hence, facilitated correct prescription of VIT.

## Antigens 5 for the Discrimination Between YJV and PDV Allergy

In Southern Europe, double sensitization to YJV and PDV is more frequently observed than that to vespid venom and HBV [34, 67, 68]. Here, a definite resolution of cross-reactivity and true primary allergy to both venoms is rarely possible due to a high degree of cross-reactivity between the major allergens of the venoms. Certainly, cross-reactivity between the antigens 5 (Ves v 5 and Pol d 5) as most relevant major allergens is of considerable importance. This was demonstrated by the fact that 17/20 (85%) of YJV-allergic patients from Germany, where PDV allergy is virtually not present, had also sIgE to Pol d 5 in ImmunoCAP™ measurements. Vice versa, 12/16 (75%) Pol d 5-reactive PDV-allergic patients from Spain (a concomitant YJV allergy cannot be fully excluded) were also reactive to Ves v 5 [32••].

Monsalve et al. demonstrated that comparing the levels of sIgE to the Ag5 allergens (Ves v 5 and Pol d 5) and phospholipases A1 (Ves v 1 and Pol d 1) allows a reliable identification of the culprit venom in 67% of double-sensitized allergic patients [30•]. A subsequent study of a very small patient cohort showed that the detection of sIgE against the same four allergens could determine the correct venom for immunotherapy in the majority, but not in all patients [69]. However, only Pol d 5 is currently available for routine diagnosis of PDV allergy on the most common sIgE singleplex platform.

To date, the gold standard to resolve double sensitization in PDV and YJV allergy is CAP-inhibition assays with PDV and YJV [31••, 70, 71]. Current limitations of the commercially available homologous allergens Pol d 5 and Ves v5 to distinguish between YJV and PDV allergy in double-positive patients by CRD were demonstrated by the fact that a good accordance between Ag5-based CRD and CAP-inhibition assays can only be achieved when the value of sIgE in kU_A_/L to Ves v 5 is about twice of those to Pol d 5 and vice versa [71, 72]. However, a later multicenter study did not find any agreement between CAP-inhibition test results and double sIgE values of Ves v 5 over Pol d 5 or vice versa [31••].

The available data demonstrates that the use of Ag5 allergens in CRD has extensive limitations in discriminating double-positivity in PDV and YJV allergy. Hence, the commercial availability of additional cross-reactive major allergen pairs (at least an addition of Pol d 1) for routine diagnosis might be beneficial for uncovering primary sensitization in PDV and YJV double-sensitized patients.

Due to the increasing spread of *Polistes dominula* on several continents [73–77], associated diagnostic problems are likely to gain importance in other areas of the world.

## Conclusions

Ag5 proteins represent a family of very potent allergens that are of major relevance in allergies to venoms of members of the Vespoidea superfamily. Although the function of Ag5 proteins in venoms remains unsolved, the study of these proteins with considerable allergenic potency may help to elucidate the molecular and immunological basis of allergenicity. Moreover, Ag5 allergens have proven to be indispensable for accurate diagnosis of venom allergy and build an essential key element for the discrimination of primary allergy and cross-reactivity in double-positivity to honeybee and vespid venom in CRD. However, the available data demonstrates that Ag5-based testing is insufficient for the differentiation between allergies to different vespid species. In this field, novel approaches, including additional allergens for CRD, are urgently needed for adequate diagnosis. Furthermore, Ag5 allergens may help to fill current diagnostic gaps such as proper diagnosis of *Polybia* venom allergy in South America [78•]. Moreover, the use of single allergens in BAT in the future might be helpful in the investigation of double-sensitized patients or in patients with a clear history of venom allergy but negative sIgE and skin tests [79].

Some studies already identified major T and B cell epitopes of Ag5 allergens or generated hypoallergenic folding variants with the aim to design novel therapeutic strategies [80–82]. However, the outstanding efficacy of the currently available VIT for Vespoidea venom allergy will make it difficult for such strategies to be realized. Nevertheless, Ag5-based therapeutics might be a practicable way for allergies to species, for which VIT is not available so far or for which substantial amounts of venom are difficult to obtain.
